# Are Hepatitis B Virus and Celiac Disease Linked?

**Published:** 2010-09-01

**Authors:** Salvatore Leonardi, Mario La Rosa

**Affiliations:** 1 Department of Pediatrics University of Catania, Catania, Italy

**Keywords:** Hepatitis B Virus, Celiac Disease, Enteropathy

## Abstract

**Background and Aims:**

It has been hypothesized that nonintestinal inflammatory diseases such as hepatitis B virus (HBV)  and hepatitis C virus (HCV) may trigger immunologic gluten intolerance in susceptible people. This hypothesis suggests a possible epidemiological link between these two diseases, although this assumption is still a matter of debate.

**Methods:**

We conducted a retrospective study to assess the prevalence of celiac disease in HBV carrier patients who had been infected in childhood.

**Results:**

None of the HBV carrier patients had immunoglobulin A antiendomysium and immunoglobulin A anti-tissue  transglutaminase, but 6 patients and 1 recovered subject had immunoglobulin A antigliadin and/or immunoglobulin G antigliadin. Moreover, no patient treated with interferon therapy showed any serological marker of celiac disease.

**Conclusions:**

Due to the small sample size, we cannot claim that there is no association between celiac disease (CD) and HBV, although in our study we did not find any CD patients. A sample size that is more representative of the prevalence of CD in Italy would better support the establishment of any possible connection between CD and HBV.

## Introduction

Celiac disease (CD), or gluten-sensitive enteropathy, is highly prevalent in genetically predisposed subjects bearing the human leulocyte antigen (HLA) DR3-DQ2 or DR4-DQ8 (prevalance rate of 1 in 200 people) [1]. The disease is characterized by malabsorption due to a local immune response to dietary gluten against the mucosa of the small intestine. In many cases the disease can be pauci-symptomatic or asymptomatic, and diagnosis is often first suggested by the appearance of associated autoimmune diseases, which are usually only found in long-standing untreated subjects [2][3][4] with severe complications [5][6]. Recently, it has been hypothesized that nonintestinal inflammatory diseases such as hepatitis B virus (HBV) and hepatitis C virus (HCV) [7][8] may trigger immunologic gluten intolerance in susceptible patients, although this assumption is still a matter of debate. Another controversial point is the possible activation of CD due to the treatment of chronic hepatitis with interferon (IFN) [9][10]. The aim of our study was to establish CD prevalence in patients with chronic HBV.

## Materials and Methods

We performed a serologic screening for glutensensitive  enteropathy in 60 subjects who had experienced HBV infection in childhood. Thirty-five  of these individuals were chronic carriers of HBV and 25 had recovered. Fifteen of the 60 subjects had used IFN-α therapy for 12 months at a dosage of   MU/m2 for the treatment of chronic HBV. All were tested for hepatitis B surface antigen (HBsAg),  antibody to hepatitis B surface antigen (anti-HBs), hepatitis B e antigen (HBeAg), antibodies to hepatits B e antigen (anti-HBe), immunoglobulin G antibody to HBc (anti-HBc IgG) and transaminase values.  Subjects were HBsAg or HBeAg positive and had high or normal transaminase values were classified as chronic HBV carriers (CHB; sex: 6M/9F; mean age: 17.8+/-2.8; median age: 18). Subjects who tested HBsAg positive, HBeAg negative, or anti-HBe positive and also had normal transaminase  values were selected as healthy HBV carriers (sex:7M/13F; mean age: 19.1+/-2.8; median age: 18.5). Finally, subjects who tested anti-HBs positive, anti-HBc IgG positive, HBeAg negative, or anti-HBe positive and had normal transaminase values (sex: 9M/16F; mean age: 19.8+/-3.7; median age:  ) were selected as having recovered from HBV. All subjects were tested for total immunoglobulin  A anti-endomysium (IgA EmA), immunoglobulin A anti-tissue transglutaminase (IgA tTGA), and immunoglobulin A and G antigliadin (IgA/IgG AGA). Sera were diluted 1:100 to determine both IgA and IgG AGA using a commercial ELISA and normal  range was considered < 10 u/ml for IgG and < 4 u/ ml for IgA. IgA EmA was examined using a monkey’s esophagus immunofluorescence assay (IFA) kit. IgA tTGA was measured using a commercial ELISA. The test was quantitative, and values greater than 7u/ml were considered positive for IgA tTGA as established by the manufacturer. Patients who tested positive for  IgG AGA but negative for IgA AGA, IgA EmA, and  IgA tTGA had a quantitative dosage of total serum IgA, which ruled out a selective IgA deficiency.

## Results

In our historical cohort study of 60 subjects with chronic hepatitis B starting in childhood, 15 had CHB,  20 were healthy carriers, and 25 were recovered. Their mean age was 21.5 +/- 2.3 years (median: 22; min: 18; max: 25), and 35 subjects (58.3%) were male. No  patients tested positive for IgA Ema or IgA tTGA. CD antibodies such as IgA AGA or IgG AGA or both were found in 7 patients (11.6%), although did not report symptoms indicative of celiac disease (Table 1). None of the subjects had selective IgA deficiency. In patients   with CHB, 3 patients were positive for IgA AGA and 2 were positive for IgG AGA. In healthy carriers, 1 was IgG AGA positive while 1 of the recovered subjects was  positive for both IgA and IgG AGA. No patient with at least one celiac-disease antibody had used IFN-α therapy in childhood. Given the high sensitivity and specificity of IgA EmA and IgA tTGA [11][12] and the  low sensitivity and specificity of IgA/IgG AGA for the diagnosis of CD, endoscopy and duodenal biopsy were not suggested to patients who were Ig AGA positive, especially because they did not exhibit symptoms of celiac disease

**Table 1:** Frequency of CD markers in patients with chronic HBV infection (Group1), healthyHBV carriers (Group 2), and those recovered from HBV infection (Group 3).

**Patients**	**IgA AGA**	**IgG AGA**	**IgA+IgG AGA**	**IgA EmA**	**IgAtTGA**
Chronic HBV patients (Group 1) n = 15	3	2	0	0	0
Healthy HBV carriers (Group 2) n = 20	0	1	0	0	0
Recovered from HBV (Group 3) n = 25	0	0	1	0	0

## Discussion

CD is a gluten-sensitive enteropathy characterized by malabsorption, abnormal small bowel structure and function, and intolerance to gluten. The disorder affects as many as 1% of people in several Western populations [13]. The HLA-DQ2 genotype is the most typical predisposition to result in the disorder, and the HLA-DQ8 genotype is the least frequent; however, only 20-50% of genetically predisposed individuals develop the disease, and even in monozygotic twins, only about 75% have the condition [13][14][15]. The background of these observations is unknown, and more information and further research might help medical professionals prevent the disease in predisposed individuals.

Recently, it was hypothesized that nonintestinal inflammatory diseases may trigger immunologic gluten intolerance in susceptible individuals, and HBV and HCV were thought to be suitable candidates. However this assumption is still a matter of debate. Relatively little data exist on the relationship between HBV and CD, although one third of the world's population (around 2 billion people) have been infected with HBV. It has been reported that the response rate to HBV vaccination in CD-infected individuals is lower (30-50%) than in the general population (4-10%) [16][17]. Moreover, the higher rate of unresponsiveness seems to relate to genetic features, especially HLA-DQ2. The class-II HLA allele usually linked to CD and other autoimmune diseases [18]. Consequently, it is also possible that HBV-like HCV may trigger immunologic gluten intolerance in genetically susceptible people and that chronic HBV segregates a higher percentage of CD patients. Bardella et al. reported a study in which the prevalence of HBsAg in CD patients was 2.5% [8], twice the rate reported in general population of Northern Italy, confirming a hypothetic link between CD and HBV. Considering the high sensitivity and specificity of IgA EmA and IgA tTGA in the diagnosis of CD [11][12], the main finding of our study is the absence of any relationship between celiac disease and chronic HBV because (a) there was no difference between the two groups of HBV carriers and the third group of recovered patients and (b) the prevalence rate of CD was 0%. In fact, although 7 of our patients tested positive for either IgA or IgG AGA or both, no patient had IgA EmA or IgA tTGA and all were healthy.

Possible activation of CD due to the treatment of hepatitis infection is another controversial point. There have been some reports indicating that autoimmune disorders such as insulin-dependent diabetes mellitus and celiac disease can develop during treatment with IFN-α for viral hepatitis because of its immune modulatory properties [19][20]. CD activation during interferon α or interferon α plus ribavirin therapy has recently been observed in HCV-positive patients [9], confirming that IFN α therapy could trigger CD in susceptible subjects during treatment. In our study, although evidence suggested that IFN-α can activate CD4T cells in the lamina propria and cause intestinal tissue damage [21], no patient treated in childhood showed any serological marker of CD at the time of the present study. Due to the small sample size we cannot claim that there is no association between CD and HBV, although in our study we did not find any instances of CD. A sample size that is more representative of the prevalence of CD in Italy should help to better examine the relationship between CD and HBV. We tentatively conclude that chronic HBV in childhood does not seem to be linked to CD, although a study population of 60 patients lacks the power to draw definitive conclusions. Moreover, although autoimmune diseases can appear or worsen during IFN therapy, we think that it is not mandatory to check for specific CD antibodies before beginning treatment and during follow up.
